# Retrospective Review of Arthroplasty Radiographs: How to Define an Adequate Radiograph

**DOI:** 10.7759/cureus.26697

**Published:** 2022-07-09

**Authors:** Ahmad Faraz, Mohammad Al-Ashqar, Shoaib Khan, Qamar Zaman, Joshua Smyth, James Parker, Nikhil Bhuskute

**Affiliations:** 1 General Surgery, Ulster Hospital, Dundonald, GBR; 2 Trauma and Orthopaedics, Yorkshire and Humber Deanery, Leeds, GBR; 3 Trauma and Orthopaedics, Whiston Hospital, Liverpool, GBR; 4 Emergency Department, Health Education, Yorkshire and Humber Deanery, Leeds, GBR; 5 Orthopaedics and Trauma, Leeds Teaching Hospitals NHS Trust, Leeds, GBR; 6 Orthopaedics and Trauma, Calderdale and Huddersfield NHS Foundation Trust, Huddersfield, GBR; 7 Orthopaedic Surgery, Calderdale and Huddersfield NHS Foundation Trust, Huddersfield, GBR

**Keywords:** clincal audit, x-ray analysis, msk radiology, hip and knee replacement, total joint replacement

## Abstract

Introduction

Adequacy of postoperative hip and knee radiographs has a direct impact on its interpretation. We undertook a quality improvement project by creating local standards to meet the arthroplasty team expectations for a satisfactory radiograph. The purposes of the study are 1. Assessment of the adequacy of radiographs according to defined criteria, and 2. Correlation of system and patient factors with inadequate radiographs.

Methods

Stage I: We conducted a single centre, retrospective audit to check the adequacy of a postoperative radiography following a total hip or knee replacement. A total of 100 radiographs were assessed against the nine criteria laid out with the consensus of orthopaedic surgeons and radiologists.

Stage II: We created a quality improvement proforma for use in the radiology department. We re-assessed 100 radiographs during the second cycle against the nine criteria to check the adequacy of hip and knee arthroplasty radiographs,

Results

Stage I: Of 100 radiographs, 51 were from the knee and 49 from the hip arthroplasty group. Sixty-nine radiographs were adequate considering overall criteria, and 31 radiographs were inadequate. The inadequacy in radiographs was related to the visibility of prosthesis, cement or relevant anatomy.

Stage II: We created a quality improvement performa for use in radiology department, highlighting the nine initial criteria. One hundred radiographs of hip and knee arthroplasty were re-assessed. Overall, 84 radiographs fulfilled the criteria of being adequate.

Conclusion

Adequacy of knee and hip arthroplasty radiographs is essential in picking up pathologies that can be missed otherwise. We present simple criteria to improve the adequacy of x-ray and prevent repetition of radiographs.

## Introduction

Radiography is relied upon to assess hip and knee arthroplasty and is usually preferred over other imaging techniques [1]. An adequate radiograph should detail implant type and position, fixation technique, zones of radiolucency and bone remodeling, soft tissue anomaly, implant wear and related bony defects and fractures [2]. Inadequate radiographs may pose challenge to a clinician in interpreting finer details, leading to missed diagnoses. McBride et al. reported that over 55,000 total hip replacements are performed in the United Kingdom every year. The critical tool for assessing hip and knee arthroplasty are radiographs reviewed during morning meetings and patient follow-up [3]. Adequate hip arthroplasty radiographs should reveal leg length discrepancies, centres of rotation, acetabular inclination, and anteversion, femoral stem position and cementation [4]. Anteroposterior and lateral views of the whole lower leg and skyline image of the patellofemoral joint, are included in post-total knee arthroplasty (TKA) radiographs. Weight-bearing reveals the real alignment, ligamentous laxity, and polyethylene wear are some of the other aspects [5]. In our practice, the arthroplasty surgeons noted the inadequate features in arthroplasty radiographs, which led to the development of criteria for adequate arthroplasty radiographs. There have been no previous studies to define an adequate arthroplasty radiograph, therefore we conducted this study to assess the adequacy of radiographs according to defined criteria and the correlation of system and patient factors with inadequate radiographs.

## Materials and methods

Primary audit

One hundred arthroplasty radiographs were analyzed. Patient demographic details can be found in Table 1. These radiographs were reviewed according to locally defined criteria for adequate postoperative radiographs. 

**Table 1:** Demographic Data

Age in Years	48 ± 14.5
Male	54
Female	46
Mean Body Mass Index	30 (range: 19.7 - 45)
Total Hip Arthroplasty (First Cycle)	51
Total Knee Arthroplasty (First Cycle)	49
Total Hip Arthroplasty (Second Cycle)	54
Total Knee Arthroplasty (Second Cycle)	46

The benchmark was defined with the input of seven Arthroplasty and three Radiology consultants. An expert consensus was reached outlining nine criteria that should be considered when assessing the adequacy of a postoperative radiograph following a total hip or total knee replacement. These are listed in Table 2. Four criteria were deemed essential, and if a radiograph did not meet one of them, it was deemed overall inadequate. The remaining five criteria were considered quality markers, but failing to meet them individually does not deem a radiograph overall inadequate. Criteria 5 was deemed essential for the knee replacement radiograph. Criterions 7 and 8 only apply to total hip replacements, as noted in Table 1. 

**Table 2:** Criteria For Adequate Hip And Knee Arthroplasty Radiograph AP: anterior posterior, GT: greater trochanter, LT: lesser trochanter

Is whole prosthesis and cement visible on both views?
Is all of relevant anatomy visible?
Is exposure satisfactory?
Are 2 views present? (AP/Lateral)
Is there obvious rotation on lateral radiograph with 15-20 degrees rotation?
Is there obvious rotation on AP with 15-20 degrees of internal rotation ?
For AP Pelvis: is Pubic Symphysis in the centre?
For AP Pelvis: are GT/LTs at equal level ?
Are there duplicates? (i-e >2 X-rays same view)

Data collection 

Four orthopaedic registrars retrospectively analyzed the initial (first) postoperative radiograph of 100 patients. Standard training was provided to all four surgeons on applying the criteria when interpreting the radiographs. For each patient's set of radiographs, a numerical value of '1' was given to each criterion failed, and '0' for each criterion satisfied. The radiographs were marked for overall satisfaction or inadequacy. The data was stored in a single Microsoft excel sheet, and patient details were anonymized.

Second cycle (re-audit)

After completing the first cycle, local guidelines based on criteria were published in conjunction with the Radiology department. Educational and feedback meetings were held with the radiographers to guide them about the importance of adequate radiographs. In conjunction with the radiography staff, the guidelines were published as a poster in the radiography rooms for future reference and education of radiographers. The radiographers collected data to evaluate factors associated with inadequate radiographs.

In the second cycle, 100 consecutive patients' radiographs were analyzed by the same four surgeons against the same criteria. However, this time radiographers were not blinded to the audit process and were tasked with collecting data to seek association between patient factors with inadequate radiographs. For the second loop, statistical analysis using point-biserial correlation scoring was performed to evaluate a correlation between system and patient factors with inadequate radiographs.

## Results

Results of primary audit

**Figure 1:**
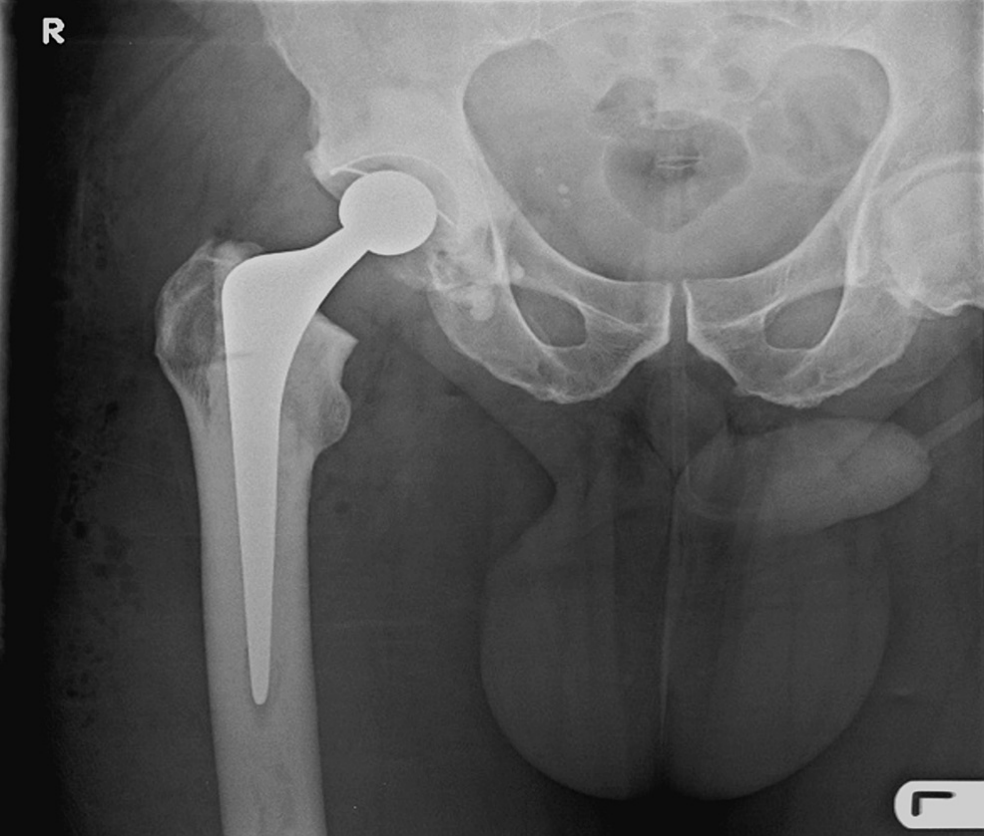
Relevant anatomy is not visible (contralateral hip)

**Figure 2:**
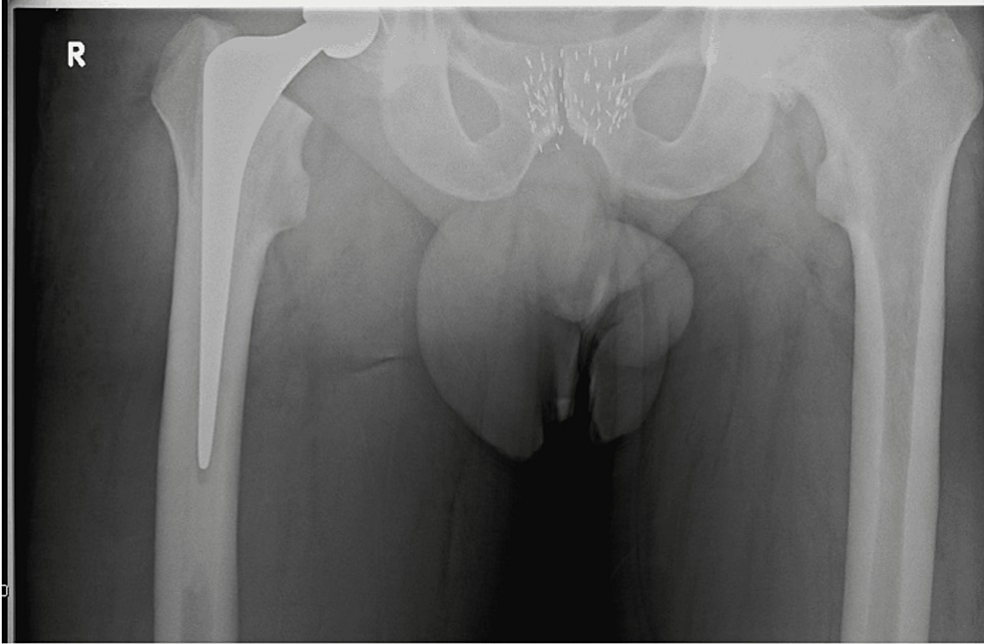
Pubic symphysis is not centred

**Figure 3:**
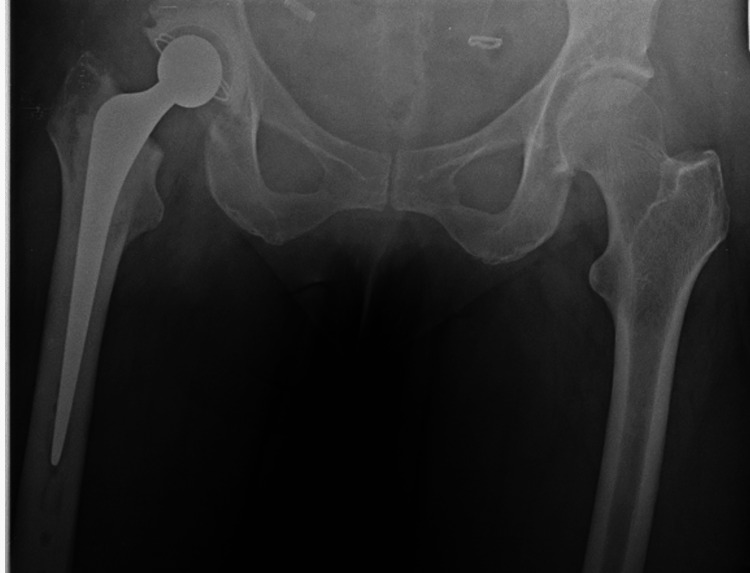
Trochanter is not levelled

**Figure 4:**
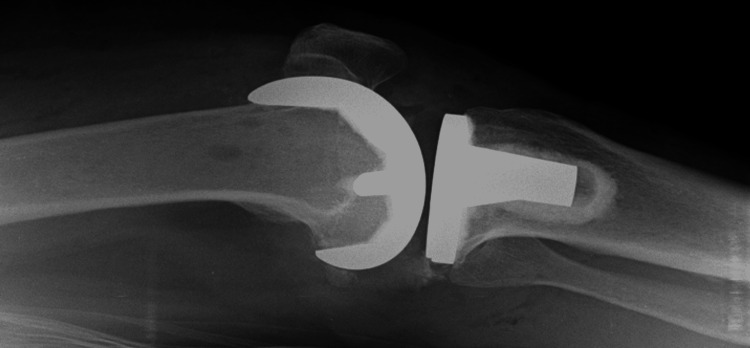
Radiograph with minimum lateral rotation

**Figure 5:**
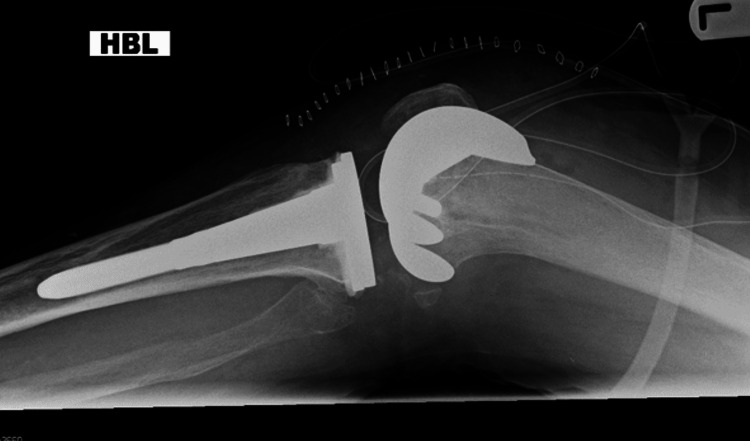
Radiograph with adequate gross lateral rotation

One hundred consecutive hip and knee arthroplasty radiographs were analysed in the first loop of the study. Of the 100 radiographs, 51 belonged to the hip and 49 to the knee arthroplasty group. There were 69 adequate radiographs considering the overall criteria, and 31 radiographs were inadequate. The inadequacy in radiographs was related to the visibility of prosthesis, cement, or relevant anatomy. Several examples of inadequate radiographs are featured in Figures 1-5.

Results of re-audit

The training of radiographers resulted in an overall improvement in the adequacy of arthroplasty radiographs which was evident in re-audit. In re-audit, 100 radiographs were evaluated for adequacy according to the criteria. Fifty-six radiographs were of hip arthroplasty and 44 of knee arthroplasty group. The re-audit showed that 84 of the hip and knee arthroplasty radiographs were adequate as per the criteria.

Hip Prosthesis

Eleven out of 56 (19.6%) total hip arthroplasty (THA) radiographs were overall inadequate. These radiographs were inadequate in meeting one or several criteria: six due to parts of the prosthesis or cement not being visible and five due to not having all relevant anatomy visible. 

Knees Prosthesis

Five out of 44 (11.4%) TKA radiographs were overall inadequate, four due to excessive rotation on the lateral view, and one due to not having the whole prosthesis visible. 

Further comparison between the first and second cycles of radiograph assessment can be reviewed in Table 3.

**Table 3:** Comparison Between Arthroplasty Radiographs During First and Second Cycles AP: anterior posterior, GT: greater trochanter, LT: lesser trochanter

Criteria	First Cycle	Second Cycle
Is whole prosthesis and cement visible on both views?	86%	93%
Is all of relevant anatomy visible?	83%	95%
Is there obvious rotation Anteroposterior view	85%	90%
Is there obvious rotation lateral view	93%	96%
Satisfactory Exposure	95%	92%
Are there duplicates? (i.e >2 X-rays same view)	79%	37%
Are 2 views present?	100%	100%
For AP Pelvis: is Pubic Symphysis in the centre?	31%	47%
For AP Pelvis: are GT/LTs at equal level?	32%	43%
Overall Satisfactory	69%	84%

Pain

Patient-reported pain scores were calculated pre- and post-radiography using the Visual Analogue Score (VAS). The mean pre-radiograph pain score was 5.46, and the post-radiograph score was 5.49, with a mean change of VAS pain score of 0.04 (range from -3 to +3). The cohort was divided into two groups for comparison: the low pain group (VAS score 1-7) and the high pain group (VAS score 8-10). The low pain group had 66 patients, nine of whom had inadequate radiographs (13.6%). The high pain group had 34 patients, seven of whom had inadequate radiographs (20.5%). When comparing all patients with point-biserial correlation, no correlation or statistically significant difference was found between VAS scores and the likelihood of an inadequate radiograph (r = -0.035, p = 0.73). 

Body mass index (BMI) 

The BMI was recorded for 63 patients. The mean BMI was 30 (19.7-45). The patients were divided into four categories according to BMI: 1. BMI <25: 17% had inadequate radiographs; 2. BMI 25-30: 10% had inadequate radiographs; 3. BMI 30-35: 21% had inadequate radiographs; 4. BMI >35: 18% had inadequate radiographs.

When comparing all 63 patients with point-biserial correlation, no correlation and no statistically significant difference was found between patient BMI and likelihood of an inadequate radiograph (r = -0.018, p = 0.89). 

Number of radiographers present

Forty-six radiographs were taken by one radiographer, 46 radiographs were taken by two radiographers, and five were served by three radiographers. The remaining three had no data recorded regarding this. 

Five out of 46 (11%) of radiographs performed by one radiographer were inadequate. Eleven out of 46 (24%) radiographs performed by two radiographers were inadequate. Zero out of the five radiographs performed by three radiographers were inadequate. 

All 97 patients were compared with point-biserial correlation, and no correlation or no statistically significant difference was found between the number of radiographers present and the likelihood of an inadequate radiograph (r = -0.083, p = 0.42). 

**Table 4:** Correlation between pain, BMI, number of radiographers and adequate radiograph

	Correlation
Pain related to x-rays	p = 0.73
Body Mass Index	p = 0.89
Number of Radiographers	p = 0.42

## Discussion

Patient radiographs play a significant role in quality control [6]. We present a study describing the adequacy of radiological exposure for hip and knee arthroplasty radiographs. Our study has demonstrated inadequacy in hip and knee arthroplasty radiographs according to the set criteria. The criteria were established by Arthroplasty surgeons and Radiologists and were considered a gold standard for our study. The study's outcome led to the training of radiographers, which improved the adequacy of radiographs, as evident by the re-audit results. This is the first study to assess the adequacy of hip and knee arthroplasty radiographs. It is essential to obtain adequate imaging to review the implantation and the surrounding bone, ensuring that the pathologies are not missed. This will avoid repeating the radiographs, adding extra time, cost and radiation exposure. 

Parker et al. evaluated the inadequacy of 1,531 pelvic radiographs across three hospitals [7]. They found that 51.9% of the radiographs were suboptimal against the European Guidelines on Quality Criteria for Diagnostic Radiographic Images (EGQCDRI) criteria. Repeat radiographs were performed in an average of 14.7% of cases. The new pathology missed on initial radiographs was detected in an average of 4% of radiographs. In hip arthroplasty radiographs, cement thickness, cement-bone, and cement-prosthesis interface play a vital role in the scrutinizing of the postoperative radiograph [8]. The current study recorded that 7% of postoperative radiographs had part of prosthesis or cement not visible. Therefore, it was not possible to comment on adequate implantation of cement and prosthesis in these radiographs. It can also pose challenges if a comparison needs to be made between immediate postoperative and follow-up radiographs. 

Mulcahy et al. proposed that the most significant radiological predictor of loosening of the stem is changes in the position of components and progressive lucency around the stem [4]. They also suggested that aseptic loosening or osteolysis can lead to implant failure. Therefore, it is essential to have a complete view of the prosthesis to identify early loosening. Their study identified that 19.6% and 11.4% of THA and TKA radiographs were inadequate, respectively. This was due to poor exposure, inadequate stem visualization, and excessive rotation of the stem on the lateral view. AP view assesses the position of the femoral stem. The tip of the stem should be in the centre of the femoral canal and neutral alignment with the longitudinal axis of the shaft [1]. Similarly, the lateral view allows evaluation of stem position in the femoral canal and adequacy of cementation. Both views are essential in a complete evaluation of hip and knee arthroplasty radiographs. In our study, the AP view was missed in one case due to patient discomfort, which could have led to missed pathologies. 

On assessing AP view, pubic symphysis (PS) should be in the center of the X-ray for the inclusion of the entire prosthesis and cement [9]. The leg length is assessed by drawing a line between the inferior border of acetabular teardrops and pelvic reference lines. Lesser trochanters are used to determine femoral references. Leg length discrepancy is assessed by measuring the perpendicular line difference between pelvic and femoral references [10]. The current study found that 16% of radiographs did not have PS in the center, whereas 23% did not show an equal distance between the lesser and greater trochanter. We further found that 22% of radiographs had similar errors in the re-audit.

VAS is a validated subjective assessment of pain. It is recorded by marking a 10 cm line representing no to maximum pain [11]. We divided the cohort into two groups based on pain score for correlation with the adequacy of radiographs. The low pain group (VAS 1-7) had 66 patients, nine of whom had inadequate radiographs (13.6%). The high pain group (VAS 8-10) had 34 patients, seven of whom had inadequate radiographs (20.5%). There was no significant difference between the groups regarding the adequacy of radiographs, showing that pain does not contribute towards inadequate radiography. 

Limitations of the study

Our study has some limitations. First, this is single-center study with a small dataset. Second, we did not evaluate the number of repeat radiographs and review them to study new findings. Despite the limitations, we were able to highlight the percentage of inadequate hip and knee arthroplasty radiographs according to established criteria. We stress the need for training to improve the adequacy of radiographs that can avoid repeating the radiographs, ultimately saving time, cost and radiation exposure. Also, adequate radiographs are more likely to guide clinicians in evaluating pathologies and follow them appropriately. 

## Conclusions

To summarise, we conducted a quality improvement project by designing local guidelines to improve the quality of arthroplasty radiographs. Adequacy of hip and knee arthroplasty is essential in picking up pathologies that can be missed with an inadequate radiograph. Educational intervention and good communication between surgeons and radiographers can improve quality of postoperative radiographs and decrease the need for repeat studies.
